# T cell Cyp27b1 promotes granzyme B expression and antiviral host defense in a mouse model of H1N1 influenza A virus infection

**DOI:** 10.3389/fimmu.2026.1944713

**Published:** 2026-10-14

**Authors:** Nicole E. Froelich, Mengzhu Tang, Nicholas J. Hanna, Troy C. Sutton, Mark B. Meyer, Margherita T. Cantorna

**Affiliations:** 1 Department of Veterinary and Biomedical Sciences, The Pennsylvania State University, University Park, PA, United States; 2 Department of Food and Nutritional Sciences, University of Wisconsin, Madison, WI, United States

**Keywords:** CD8+ T cells, Cyp27b1 1α-hydroxylase, granzyme B, influenza A virus, vitamin D

## Abstract

**Introduction:**

Immune cells express vitamin D receptors (VDR) and the CYP27B1 enzyme needed to produce the VDR ligand, 1,25(OH)_2_D; suggesting that perhaps the vitamin D response is regulated locally at the site of infection. Here, we examined the role of immune cell-derived 1,25(OH)_2_D in host resistance to influenza infection.

**Methods:**

Mice were infected with mouse-adapted H1N1 influenza A virus, to determine the role of CYP27B1 on host resistance to influenza infection. The mouse strains infected include; wild type (WT) littermates, whole-body Cyp27b1 knockout (Cyp27b1 KO) mice, mice lacking renal 1,25(OH)_2_D production (double intronic (DI)KO), mice with T cell-specific deletion of Cyp27b1 (T-Cyp27b1 KO), and mice with T cell-specific deletion of Vdr (T-Vdr KO).

**Results:**

Whole body Cyp27b1 KO mice developed severe influenza A virus disease, including infection induced mortality, compared to WT littermates. Oral treatments of the Cyp27b1 KO mice with 1,25(OH)_2_D failed to completely protect the Cyp27b1 KO mice from influenza A virus disease. Mice without renal 1,25(OH)_2_D had similar influenza disease as WT littermates. Influenza infection of T cell-specific Cyp27b1 KO resulted in increased respiratory distress and lethargy compared to WT littermates. WT and DIKO mice had more CD8+ T cells expressing higher amounts of granzyme B+ (GrzB) and disruptions in PD-1 than either whole-body Cyp27b1 KO or T-Cyp27b1 KO mice.

**Discussion:**

The work suggests that renal 1,25(OH)_2_D is not required for host resistance to this influenza A virus infection. The work further demonstrates that immune cells and in particular T cells may produce 1,25(OH)_2_D to locally support CD8+ T cell responses to influenza A virus infection. Defining the mechanisms that regulate local 1,25(OH)_2_D production in T cells or other immune cell types is needed.

## Graphical Abstract

**Figure:**
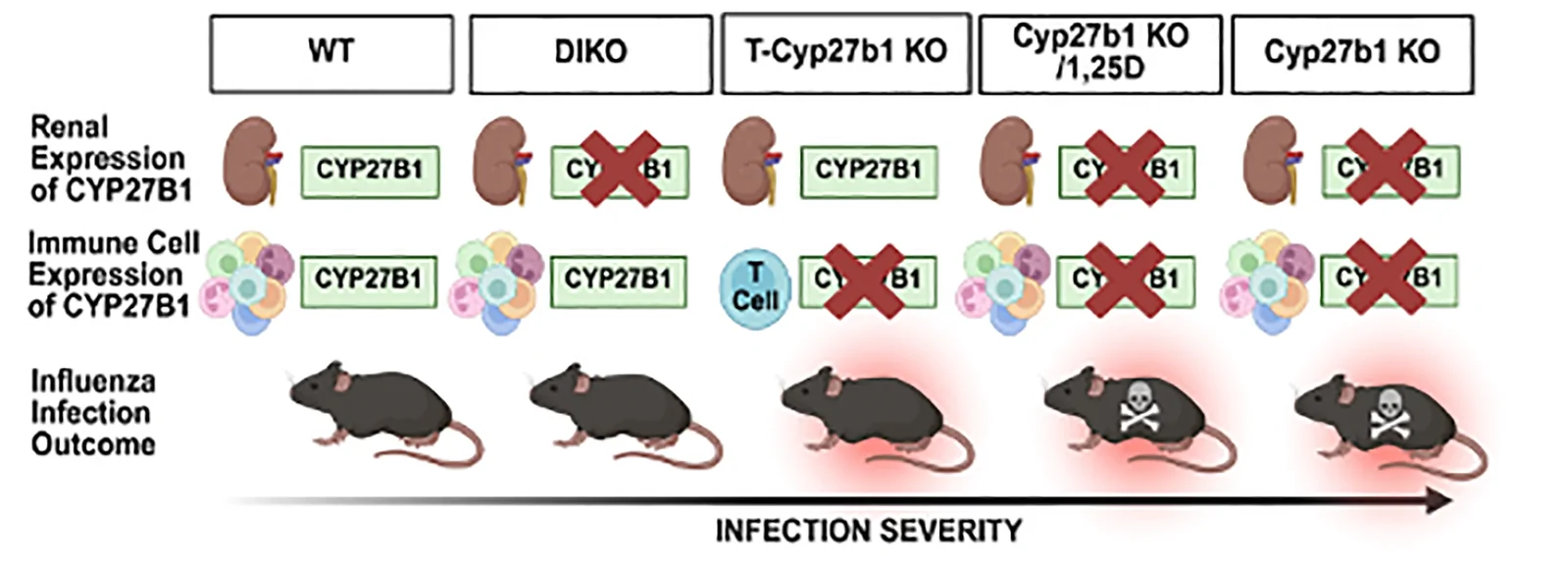
Extra-renal CYP27B1 protects against severe influenza A virus disease. Deletion of CYP27B1 in T cells or in the whole-body worsens influenza A disease severity in mice, whereas deletion of renal CYP27B1 does not. Oral 1,25(OH)(1,25D) did not completely recover the lethal disease in Cyp27b1 KO mice. Together the data suggests that local immune cell production of 1,25(OH)(1,25D) by CYP27B1, including by T cells, is important for host immunity to influenza.

## 1 Introduction

Vitamin D sufficiency has been associated with protection from acute respiratory infection in humans (1). Vitamin D is inactive and must undergo two hydroxylations to produce the biologically active 1,25(OH)_2_D. *CYP27B1* encodes the 1-alpha hydroxylase enzyme responsible for producing 1,25(OH)_2_D, which is the high-affinity vitamin D receptor (VDR) ligand (2, 3). The main source of CYP27B1 is in the kidney, where the hormones fibroblast growth factor (FGF)-23 and parathyroid hormone (PTH) control its expression (4–6). Immune cells express the VDR and are therefore vitamin D targets (7–9). Immune production of CYP27B1 is not regulated by PTH and FGF-23; instead, activation of immune cells induces production of 1,25(OH)_2_D (5, 10). The role of local production of 1,25(OH)_2_D by immune cells in host resistance to influenza A virus infection has not been investigated.

The severity of infection is determined by several factors including the host immune response to the influenza A virus. In particular, antigen specific CD8+ cytotoxic T lymphocytes are critical for viral clearance (11). Cytotoxic CD8+ T cells recognize and kill influenza virus-infected cells through the release of cytotoxic granules containing perforin and granzyme B (GrzB) (12). During influenza infection, cytotoxic GrzB+ and IFN-γ producing CD8+ T cells predominate at the site of murine infection in the lung. IFN-γ production upregulates antiviral genes in nearby cells, recruits a pro-inflammatory response to control viral replication, and contracts the CD8+ T cell response as infection clears (13). When this T cell response is insufficient or dysregulated, viral clearance is impaired, leading to increased inflammation and severe lung pathology.

Vitamin D deficiency has been shown to result in excess inflammation following viral respiratory infection (14, 15). Vitamin D deficient mice developed more severe influenza A virus infection, with overproduction of inflammatory cytokines and more immune cell infiltrates in the lungs than vitamin D sufficient mice (14). Vitamin D inhibits the proliferation of T cells and reduces proinflammatory cytokine production (16–18). Vdr KO mice had CD8+ and CD4+ T cells that overproduced IFN-γ and IL-17 (19–22). Activated T cells from mice and humans have been shown to express CYP27B1 (23–25). Decreased expression of GrzB was shown in CD8+ T cells from Vdr KO mice infected with lymphocytic choriomeningitis although the effect of lower GrzB in CD8+ T cells on disease severity was not shown (26). The effect of local vitamin D regulation and CYP27B1 on the CD8+ T cell response to influenza A virus infections has not been studied.

A series of genetic knockout models were used to examine the systemic, local, and T cell-intrinsic production of 1,25(OH)_2_D in the host response to influenza A virus infection. We previously showed that Cyp27b1 KO mice developed a lethal influenza virus infection, while all WT littermates survived the infection (14). Cyp27b1 KO mice had fewer GrzB+ CD8+ T cells and reduced expression of PD-1 following *in vitro* activation than CD8+ T cells from WT mice. Oral 1,25(OH)_2_D treatments of Cyp27b1 KO mice did not restore GrzB+ CD8+ T cell expansion and the Cyp27b1 KO/1,25(OH)_2_D treated mice had intermediate clinical signs that were less severe than Cyp27b1 KO mice and more than WT mice infected with influenza A virus. Mice without renal CYP27B1 had similar clinical disease signs as WT mice following influenza A virus infection. Conversely, T cell deletion of CYP27B1 resulted in more severe influenza A virus disease demonstrating an *in vivo* role for T cell expression of CYP27B1 in regulating local immunity. Lower frequencies of GrzB+CD8+ T cells and less GrzB+ per cell were shown in the lung of Cyp27b1 KO and T-Cyp27b1 KO mice compared to their WT littermates. Collectively, these studies indicate CYP27B1 production by T cells is important for an optimal GrzB+ response and for controlling disease severity following influenza A virus infection with this H1N1 virus.

## 2 Methods

### 2.1 Mouse models and diets

C57BL/6 WT, Cyp27b1 KO, M1/M21-double intronic (DI)KO (10) and Cyp27b1^fl/fl^ (Dr. Mark Meyer, University of Wisconsin, Madison, WI), Lck-cre (B6.Cg-Tg(Lck-cre)548Jxm/J), Jackson laboratories, Bar Harbor, ME), and Vdr^fl/fl^ mice were bred and housed at the Pennsylvania State University. Mice were bred and housed under standard laboratory conditions of 12-hour light/dark cycle, in a controlled temperature and humidity environment with ad libitum access to diet and water. All experiments used 8–10 wk old, randomly selected and sex matched male and female mice of each genotype. Experiments were not powered to determine sex as a variable which is a limitation of the study. Cyp27b1 KO and DIKO mice and their WT littermates were maintained on vitamin D containing high calcium, high phosphate rescue diet (20% Lactose, 2.0% Ca, 1.25% P, TD.96348, Envigo, Madison, WI) to normalize serum calcium and PTH levels to that of WT mice. All other experiments that used T-Vdr KO or T-Cyp27b1 KO and WT littermates used the same vitamin D containing diets but without the high-lactose (Envigo, TD 89124). Cyp27b1 KO, Cyp27b1^fl/fl^, Vdr^fl/fl^, and Lck-cre mice were genotyped via ear clip DNA. Genotyping primer sequences can be found in **Supplementary Table 1**. For all experiments mice were fed the same vitamin D sufficient diets (as their WT littermate controls.

In one experimental design, Cyp27b1 KO mice received 200 ng of 1,25(OH)_2_D injected intraperitoneally on d0 of infection and then orally dosed with 20 ng of 1,25(OH)_2_D in corn oil beginning on d1 post-infection and continuing daily for the rest of the experiment. Serum was collected to monitor hypercalcemia, which was not observed at these doses of 1,25(OH)_2_D. All animal studies were approved by the Institutional Animal Care and Use Committee (IACUC) Pennsylvania State University (University Park, PA).

### 2.2 Influenza A virus infection

Mice were anesthetized with isoflurane and intranasally inoculated with 30–50 TCID50 mouse-adapted A/California/04/2009 (pdmH1N1) in 50μL Gibco’s reduced-serum minimum essential media (ThermoFisher, Waltham, MA, USA) (27). Mice were monitored daily for survival, body weight loss, and clinical signs [scored blinded on a scale of 0-3, where 0 is equivalent to no symptoms, and 3 is distressed, showing labored breathing and reduced movement in response to stimuli (14)] for up to 14 days post-infection. Mice reaching predetermined humane endpoints (extreme respiratory distress and/or 25% body weight loss) were sacrificed using carbon dioxide exposure (IACUC approved) and recorded as having succumbed to the infection (14).

### 2.3 Tissue cell isolation

Spleen tissue was mechanically homogenized in 1X PBS and filtered through a 70 µm cell strainer. Lung tissue was minced and digested in Ca2+/Mg2+ free Hanks balanced salt solution (HBSS) containing 1 mM 1,4-dithiothreitol and 10 mM EDTA at 37 °C for 30 minutes, and then in a RPMI-c media containing collagenase type I (300 U/mL) and DNase I for 1 hour at 37 °C. Cells were filtered through a 70 µm cell strainer. Cell suspensions from all tissues were then treated with red blood cell lysis buffer and resuspended in PBS with 5% FBS.

### 2.4 Flow cytometry and CFSE proliferation

Spleen cells were resuspended in 1XPBS and treated with CFSE diluted in DMSO for 10 minutes at 37 °C. 1X PBS with 5% FBS was added to quench the CFSE, and cells were washed. Cells were then washed with PBS/FBS three times, counted and plated in RPMI-C. Splenocytes were cultured at 2 million per 1 mL media onto anti-CD3e coated plates for 48h and 72h. Supernatants and CFSE stained cells were collected. CFSE stained cells were gated on lymphocytes, singlets, CD3e+ cells, and then CD4+CD8-, and CD8+CD4- subsets. Within these subsets. CFSE was plotted via histogram against a non CFSE treated and a CFSE treated but not stimulated control to determine proliferation.

For intracellular staining, single cell suspensions were treated with 2 µg/mL Brefeldin A in RPMI-c for 4 hours at 37 °C, 5% CO_2_. Extracellular staining was done in PBS with 5% FBS for 20 minutes at 4 °C, followed by a 10-minute fixation with 2% paraformaldehyde. Cells were resuspended in fixation/permeabilization buffer for 30 minutes at room temperature and intracellularly stained in permeabilization buffer at 4 °C overnight. Samples were washed and resuspended in PBS with 5% FBS to be processed on the Cytek Aurora, a spectral flow cytometer at the PennState Flow Core Facility. Samples were analyzed with FlowJo version 10.8. Unstained controls and compensation beads were included for each experiment. **Supplementary Figure 1** has the gating strategies for the GrzB+ and PD-1 expressing CD4+ and CD8+ T cells. All flow cytometry markers are listed in **Supplementary Table 2**.

### 2.5 Serum 25(OH)D and IFN-γ measurements

Serum 25(OH)D was measured via ELISA kit and standards as per the manufacturer’s instructions (Eagle Biosciences, Amherst, NH). IFN-γ was measured by ELISA with manufacturer issued standards and kit (BD Biosciences, San Diego, CA). The limits of detection were 1.6 ng/ml for 25(OH)D and 2 pg/mL for IFN-γ.

### 2.6 RNA isolation and quantitative PCR

T Cells were isolated via magnetic isolation kit (EasySep Mouse T-cell Isolation Kit) from the spleen of T-Cyp27b1 KO mice and WT littermate controls for validation of *Cyp27b1* deletion via PCR of cDNA. Lung, kidney and splenic T cells were collected in Trizol reagent and stored at -80 °C until processing. All tissue and cell samples were homogenized, and RNA was extracted by chloroform-isopropanol precipitation. RNA was quantified by nanodrop and normalized to 2 µg for reverse transcription. Quantitative PCR was performed using SYBR green mix and primers from Integrated DNA Technologies as listed in **Supplementary Table 3**. Gene expression for *Cyp24a1* was analyzed via the ΔΔCt method relative to housekeeping *Gapdh* and normalized to non-1,25(OH)_2_D treated controls. Viral H1N1 matrix (*M) gene* was quantified as fold change relative to housekeeping *Gapdh* and normalized to uninfected WT controls. Validation of gene deletion of *Cyp27b1* was quantified via the ΔΔCt to GAPDH and normalized to the values from isolated T cells from WT littermates. GAPDH stability was verified by standard deviations of <0.5 across genotypes, genes and infection time points and was used as the only reference gene for qPCR normalization. Melt curve analysis was ran to confirm amplification specificity.

### 2.7 Statistical analysis

Data was graphed using GraphPad Prism. Choice of statistical test was determined by the number of groups analyzed, sample size, and data distribution/normality. For simple pairwise or multi-group comparisons, normality was assessed using the Shapiro-Wilk test, and T test, one- or two-way ANOVA was selected accordingly. For Two-way ANOVA of flow cytometry data sets, Tukey’s multiple comparison test was used. For clinical scores that measured repeatedly in the same animals across the infection time course, two-way ANOVA or mixed-effects analysis was used, with day post-infection treated as a repeated factor to determine Day × Genotype interaction over the course of infection. Mixed effects analysis was applied where data were incomplete due to humane endpoint euthanasia. Normality for these repeated-measures models was evaluated by QQ plot of model residuals, which approximated a normal distribution with minor deviation at the extreme tails, supporting the appropriateness of parametric analysis and the reporting of data as mean ± SEM for these outcomes. Survival was reported via the log-rank survival test. Given the large number of comparisons generated across the 14-day time course, multiple comparisons were corrected using the two-stage step-up method of Benjamini, Krieger, and Yekutieli to control the false discovery rate. Outlier analysis was performed for each dataset using the ROUT method (Q = 1%). Values are expressed as mean ± SEM. Significance threshold is p <0.05.

## 3 Results

### 3.1 Cyp27b1 KO T cells proliferate faster and produce more IFN-γ than WT T cells

*In vitro*, 1,25(OH)_2_D has been shown to inhibit T cell proliferation and IFN-γ production (28). Similarly, T cells that are VDR negative proliferate faster than those that do express the VDR (19, 20). The proliferative ability of T cells from Cyp27b1 KO mice was determined using CFSE dilution assays. CD8+ T cells from Cyp27b1 KO mice proliferated significantly more than WT CD8+ T cells at 48h (**Figure 1A**) and 72h (**Figure 1B**). CD4+ Cyp27b1 KO T cells showed significantly increased proliferation at 72h, but not at 48h (data not shown), as compared to WT CD4+ T cells (**Figure 1B**). T cells from Cyp27b1 KO mice proliferated more than T cells from their WT littermates (**Figure 1**). In addition, the T cells from Cyp27b1 KO mice produced significantly more IFN-γ than T cells from WT mice (**Figure 1C**). Although the amount of 1,25(OH)_2_D was not measured directly here, prior work has shown activated CD4+ T cells from humans produce 1,25(OH)_2_D (100 pM measured by enzyme immune-assay (25), activated mouse CD4+ and CD8+ T cells express *Cyp27b1* mRNA (29) and mouse CD8+ T cells express a CYP27B1 reporter 48h post-stimulation (23). Based on the literature it can be inferred that the activated T cells in the WT cultures (**Figure 1**), but not the Cyp27b1 KO cultures, produce 1,25(OH)_2_D that acts on the T cells to suppress proliferation and reduce IFN-γ production. The reduction in IFN-γ and suppression of T cell proliferation are in line with the well described exogenous effects of adding 1,25(OH)_2_D to activated mouse and human T cells in culture (30–32).

**Figure 1:**
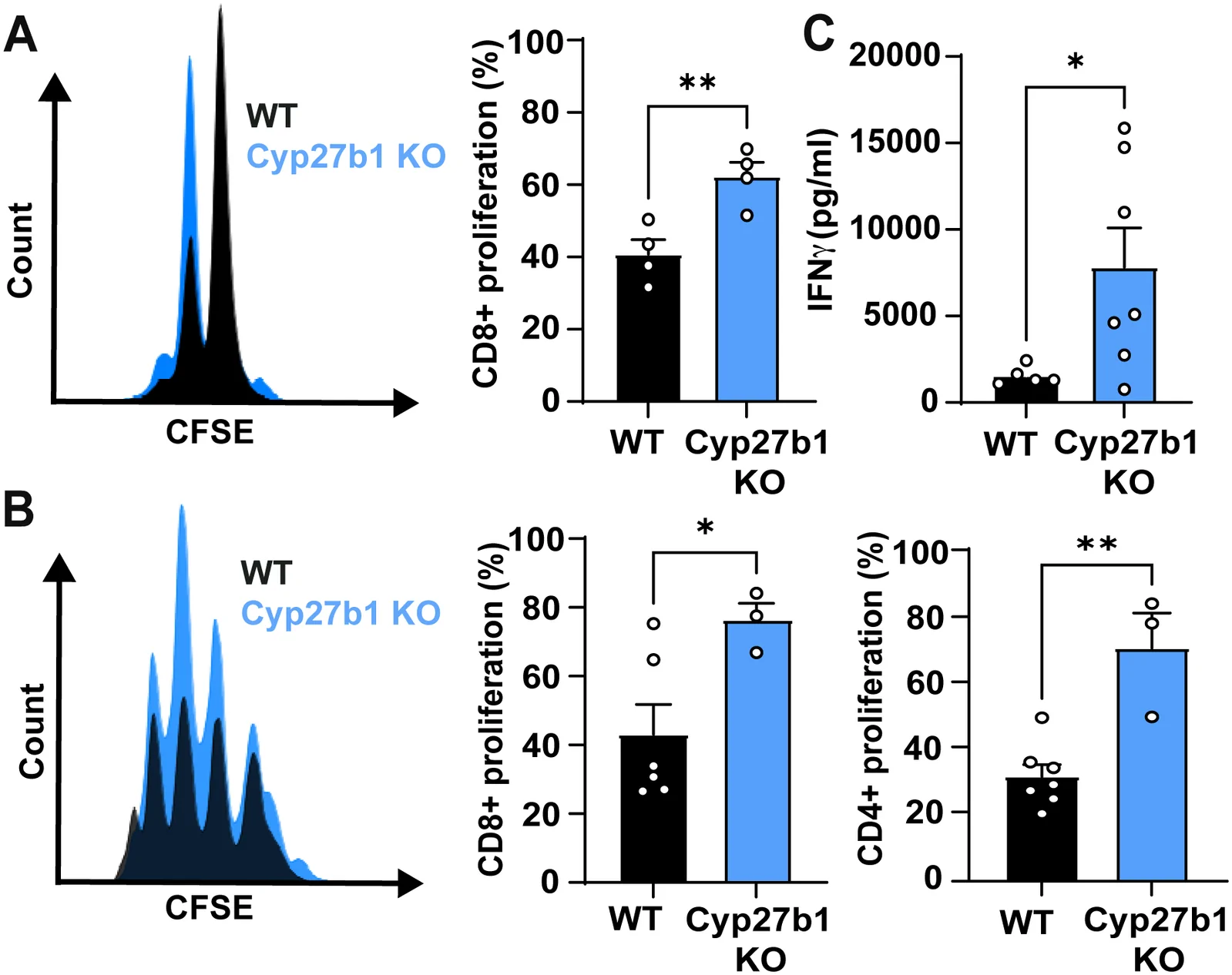
T cells from Cyp27b1 KO mice proliferate more and make more IFN-γ than WT T cells. Representative CFSE dilution histograms for WT (black) and Cyp27b1 KO (blue) T cells stimulated with anti-CD3 for 48h, n=4 mice of each genotype **(A)** or 72h, n=3–6 mice of each genotype **(B)**. **(C)** IFN-γ production at 72h post-stimulation, n=3–7 mice of each genotype. Each symbol represents an individual mouse per timepoint, and results are from 3 independent experiments. Values are the mean ± SEM. Statistical significance was determined by unpaired t-test, *p < 0.05, **p < 0.01.

### 3.2 Cyp27b1 KO mice have more severe influenza A virus disease and fewer CD8+/GrzB+ T cells

Confirming our previous data, Cyp27b1 KO mice developed significantly more severe clinical signs after influenza A virus infection than their WT littermates (**Supplementary Figure 2**) (14). Forty percent of the Cyp27b1 KO mice had to be sacrificed before d14 post-infection due to severe influenza A virus clinical signs as compared to no lethality in WT mice (**Supplementary Figures 2A–D**). Weight loss developed earlier and was significantly higher in Cyp27b1 KO than in the WT littermates (**Supplementary Figure 2B**). Viral *M gene* expression was significantly higher in the lung of Cyp27b1 KO mice than WT littermates at d3 post-infection (**Supplementary Figure 2E**).

The immune cell infiltrates in the spleen and lung from Cyp27b1 KO and WT littermates were evaluated. PD-1 expression acts as a marker for T cell activation as well as an immune checkpoint to repress activated T cells (33, 34). *In vitro*, activation of splenic T cells resulted in induction of PD-1 expression in both WT and Cyp27b1 KO T cells (**Figure 2A**; **Supplementary Figure 3A**). At 48h, but not 72h, post-stimulation the CD8+ T cells from WT mice had higher frequencies of CD8+/PD-1+ than Cyp27b1 KO mice (**Figure 2A**). In CD4+ T cells PD-1+ expression in Cyp27b1 KO splenocytes was not significantly different than WT (**Supplementary Figure 3A**). In the lung tissue of uninfected Cyp27b1 KO mice *Pd-1* mRNA expression was significantly decreased compared to the lung tissue of WT littermates (**Figure 2B**). The frequencies of CD4+ and CD8+ T cells in the lung of WT and Cyp27b1 KO mice were not different between genotypes or with influenza A virus infection (**Supplementary Figures 3D, E**). Influenza A virus infection of WT mice showed an increase in PD-1 expression on both CD4+ and CD8+ T cells in the spleen at d6 post-infection (**Supplementary Figure 3B**; **Figure 2C**) and in the lung at d6 and d9 post-infection (**Supplementary Figure 3C**; **Figure 2D**). Conversely, influenza A virus infection had no effect on PD-1 expression on CD4+ and CD8+ T cells in the spleen of Cyp27b1 KO mice (**Supplementary Figure 3B**; **Figure 2C**). There were significantly lower frequencies of CD8+/PD-1+ T cells in the Cyp27b1 KO spleen at d6 post-infection compared to the WT littermates (**Figure 3C**). The MFI for PD-1 expression on CD8+ and CD4+ T cells was not different in the lung between Cyp27b1 KO and WT (**Figure 2E**; **Supplementary Figure 3F**). PD-1 MFI was significantly higher on both CD4+ and CD8+ T cells at d6 post-infection in the spleen (**Supplementary Figure 3G**; **Figure 2F**). Influenza A virus infection induced the expansion of GrzB+ T cells in the lung (**Figure 2G**; **Supplementary Figure 3H**). WT mice had significantly higher CD8+/GrzB+ frequencies and GrzB MFI prior to infection (d0) and at d6 post-infection than Cyp27b1 KO mice (**Figures 2G, H**). Compared to CD8+ T cells, CD4+ T cells are not a significant source of GrzB (lower frequencies and lower MFI, **Supplementary Figure 3**). PD-1 and GrzB expression are differentially expressed on Cyp27b1 KO and WT T cells following influenza A virus infection.

**Figure 2:**
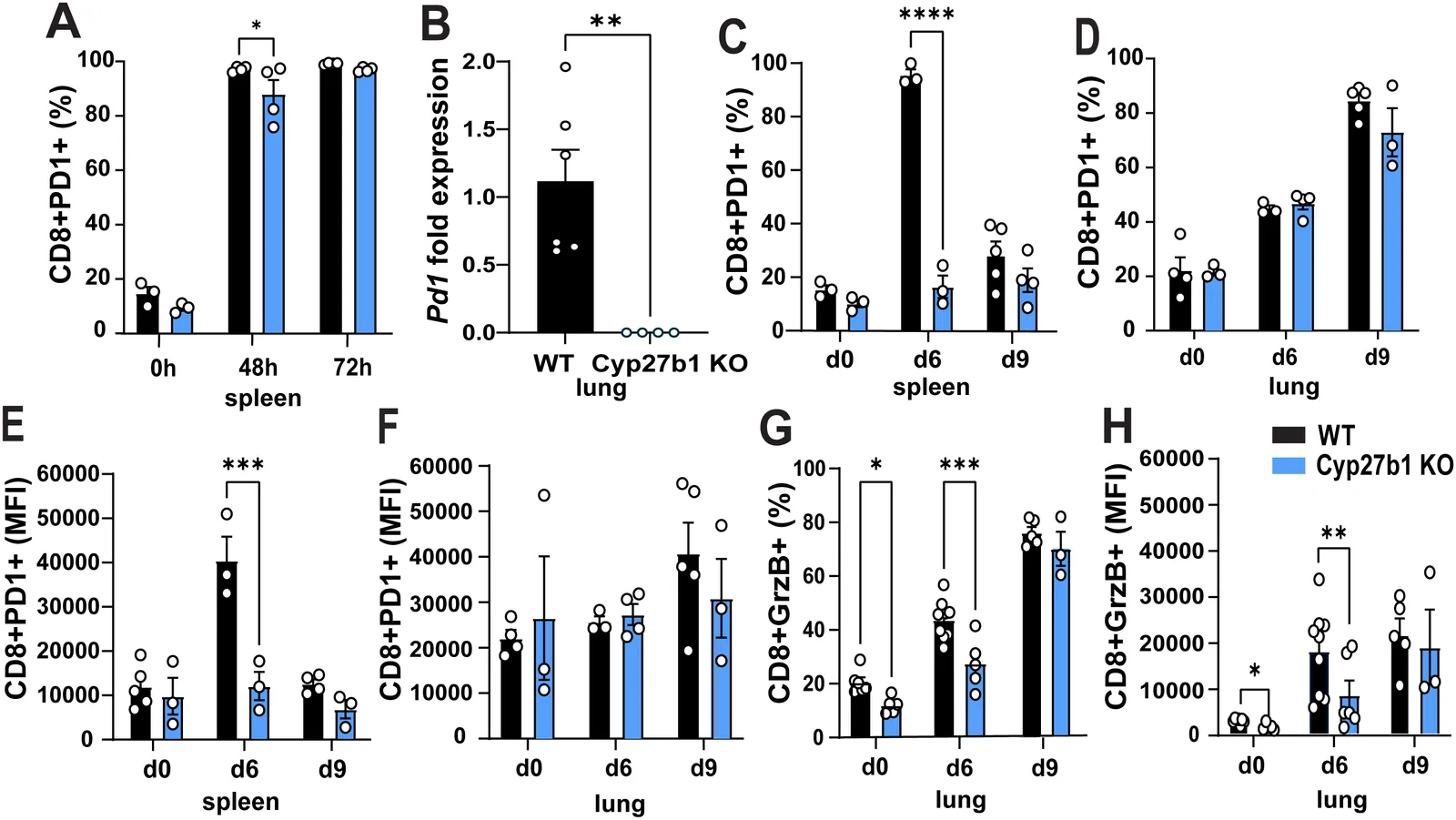
PD-1 and GrzB expression in CD8+ T cells are affected by Cyp27b1. **(A)** Frequency of CD8+/PD-1+ T cells following anti-CD3 stimulation *in vitro*. **(B)** *Pd-1* mRNA expression in the d0 lung. **(C, D)** Frequency of CD8+/PD-1+ T cells in the **(C)** spleen and **(D)** lung. Mean fluorescence intensity (MFI) of PD-1+ in CD8+ T cells from the **(E)** spleen and **(F)** lung. Frequency of **(G)** CD8+/GrzB+ T cells and **(H)** GrzB MFI in CD8+ T cells from the lung. WT (n= 3-8) and whole body Cyp27b1 KO (n=3-6) mice for each time point. Each symbol represents an individual mouse per timepoint. **(A, B)** are reflective of two individual experiments and **(C–H)** include pooled results from 4 individual experiments. Values are the mean ± SEM. Statistical significance was determined using **(B)** unpaired Student’s t-test and **(A, C–H)** two-way ANOVA with Tukey correction (multi-group/timepoint comparisons), *p < 0.05, **p < 0.01, ***p < 0.001, ****p < 0.0001.

**Figure 3:**
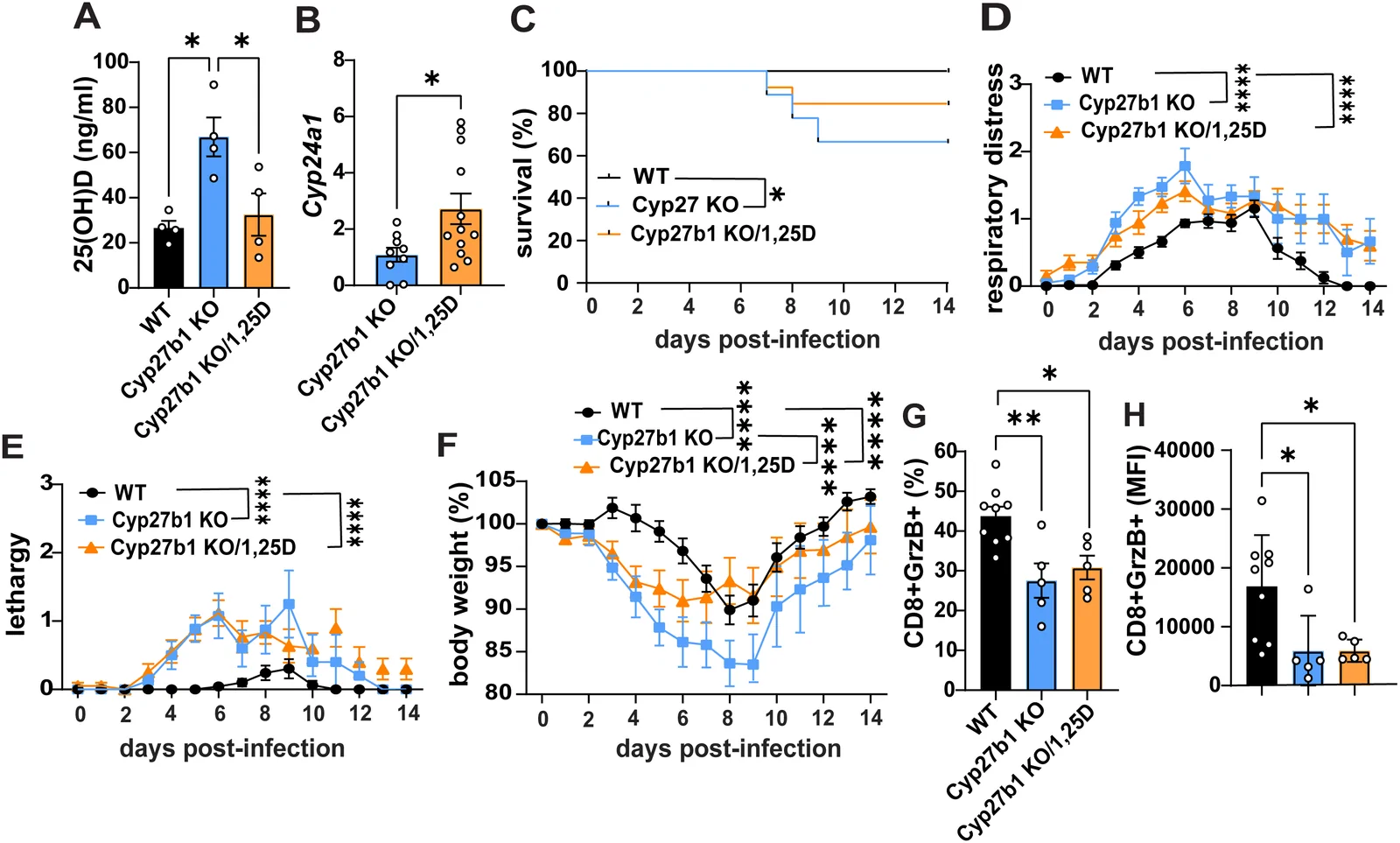
1,25(OH)_2_D treatment of Cyp27b1 KO mice is ineffective to support GrzB expression in CD8+ T cells or to protect from influenza A virus disease. **(A)** Serum 25(OH)D levels (ng/mL) of WT, Cyp27b1 KO, and 1,25(OH)_2_D (1,25D) dosed Cyp27b1 KO (Cyp27b1 KO/1,25D) (n=4 mice per group, and 2 individual experiments). **(B)** *Cyp24a1* mRNA expression in whole lung tissue of Cyp27b1 KO (n=9) and Cyp27b1 KO/1,25D (n=12) mice, and data pooled from 3 experiments. **(C)** Survival, **(D)** respiratory distress, **(E)** lethargy, and **(F)** bodyweight loss following influenza A virus infection of WT (n=34), Cyp27b1 KO (n=21), and Cyp27b1 KO/1,25D (n=20) from 9 individual experiments. **(G)** Frequency of CD8+/GrzB+ T cells and **(H)** GrzB MFI in CD8+ T cells in the lung at d6 post-infection from WT (n=8), Cyp27b1 KO (n=4) and Cyp27b1 KO/1,25D (n=5) from 4 independent experiments. Values are the mean ± SEM. Significance was determined using **(A, B)** unpaired Student’s t-test, **(C)** was analyzed by Mantel-Cox log-rank test. **(D–H)** were analyzed by two-way ANOVA with Tukey’s multiple comparisons test and were adjusted using the two-stage step-up Benjamini, Krieger, and Yekutieli FDR for multiple comparisons *p < 0.05, **p < 0.01, ***p < 0.001, ****p < 0.0001.

### 3.3 1,25(OH)_2_D treatments fail to completely protect Cyp27b1 KO mice from influenza A virus disease

CYP27B1 mediated production of 1,25(OH)_2_D results in a feedback loop that induces the vitamin D 24-hydroxylase (CYP24A1), which targets both 1,25(OH)_2_D and 25(OH)D for degradation (2). Feeding Cyp27b1 KO mice a vitamin D sufficient diet results in the accumulation of 25(OH)D in the serum, that is significantly higher than in WT mice (**Figure 3A**) (35). Confirming the *in vivo* bioactivity of the 1,25(OH)_2_D treatments, renal expression of *Cyp24a1* was significantly increased by the 1,25(OH)_2_D dosing of Cyp27b1 KO mice (**Figure 3B**). In addition, the 1,25(OH)_2_D treatments reduced 25(OH)D levels in the Cyp27b1 KO mice to those in WT mice, demonstrating the effectiveness of the 1,25(OH)_2_D treatments for controlling 25(OH)D levels (**Figure 3A**). Influenza A virus infection of the Cyp27b1 KO/1,25(OH)_2_D treated mice resulted in less lethality than the lethality in Cyp27b1 KO mice and more lethality than WT mice (**Figure 3C**), however only the WT and Cyp27b1 KO survival curves were significantly different. This intermediate phenotype of the Cyp27b1 KO/1,25(OH)_2_D mice between the untreated Cyp27b1 KO and WT mice was also evident in the body weight loss changes, respiratory distress, and lethargy signs following influenza A virus infection (**Figures 3D–F**). However, 1,25(OH)_2_D treatments of Cyp27b1 KO mice did not restore the frequency of CD8+/GrzB+ or GrzB MFI in T cells to WT levels found in the lung at d6 post-infection (**Figures 3G, H**). 1,25(OH)_2_D treatments of Cyp27b1 KO mice induced *Cyp24a1*, and reduced serum 25(OH)D but were ineffective for supporting GrzB expression in CD8+ T cells resulting in an intermediate phenotype of the Cyp27b1/1,25(OH)_2_D treated mice following infection with influenza A virus.

### 3.4 Renal production of Cyp27b1 is not essential for the effects of vitamin D on host resistance to influenza A virus

The defining paradigm of vitamin D immunobiology is that renal 1,25(OH)_2_D regulates immunity. Renal expression of CYP27B1 is controlled by PTH and FGF-23 (5, 10). Knocking out the two intronic enhancers that control the response of CYP27B1 to PTH and FGF-23 (double intronic knockout, DIKO) results in mice without renal production of CYP27B1, but with intact extra-renal CYP27B1 (5, 10, 29). DIKO mice still possess the extra-renal capacity to produce CYP27B1, which helps to preserve mineral homeostasis without renal 1,25(OH)_2_D (5, 10, 29). DIKO mice and their WT littermates were infected with influenza A, to determine the relative role of extra-renal 1,25(OH)_2_D production on host immunity. There was no lethality in either the DIKO or WT mice following influenza A virus infection. The kinetics and extent of body weight loss was similar in WT and DIKO mice however DIKO mice had significantly more weight loss over the course of influenza A virus infection than WT mice (**Figure 4A**). Respiratory distress and lethargy scores were not different in WT and DIKO mice infected with influenza A virus (**Figures 4B, C**). Frequencies of CD8+ and CD4+ T cells in the lung increased with infection but were not different between WT and DIKO mice (**Supplementary Figures 4A, B**). Influenza A virus infection increased PD-1 on CD4+ and CD8+ T cells and GrzB in CD8+ T cells in the lung between d6 and d9 post-infection in both DIKO and WT mice (**Figures 4D–G**; **Supplementary Figures 4C, D**). Splenic PD-1+ expression on CD4+ and CD8+ T cells was higher at d6 than d9 post-infection in both DIKO and WT mice (**Figures 4H, I**; **Supplementary Figures 4E, F**). Renal expression of CYP27B1 is not required for GrzB production in CD8+ T cells or for an effective host response to influenza A virus infection.

**Figure 4:**
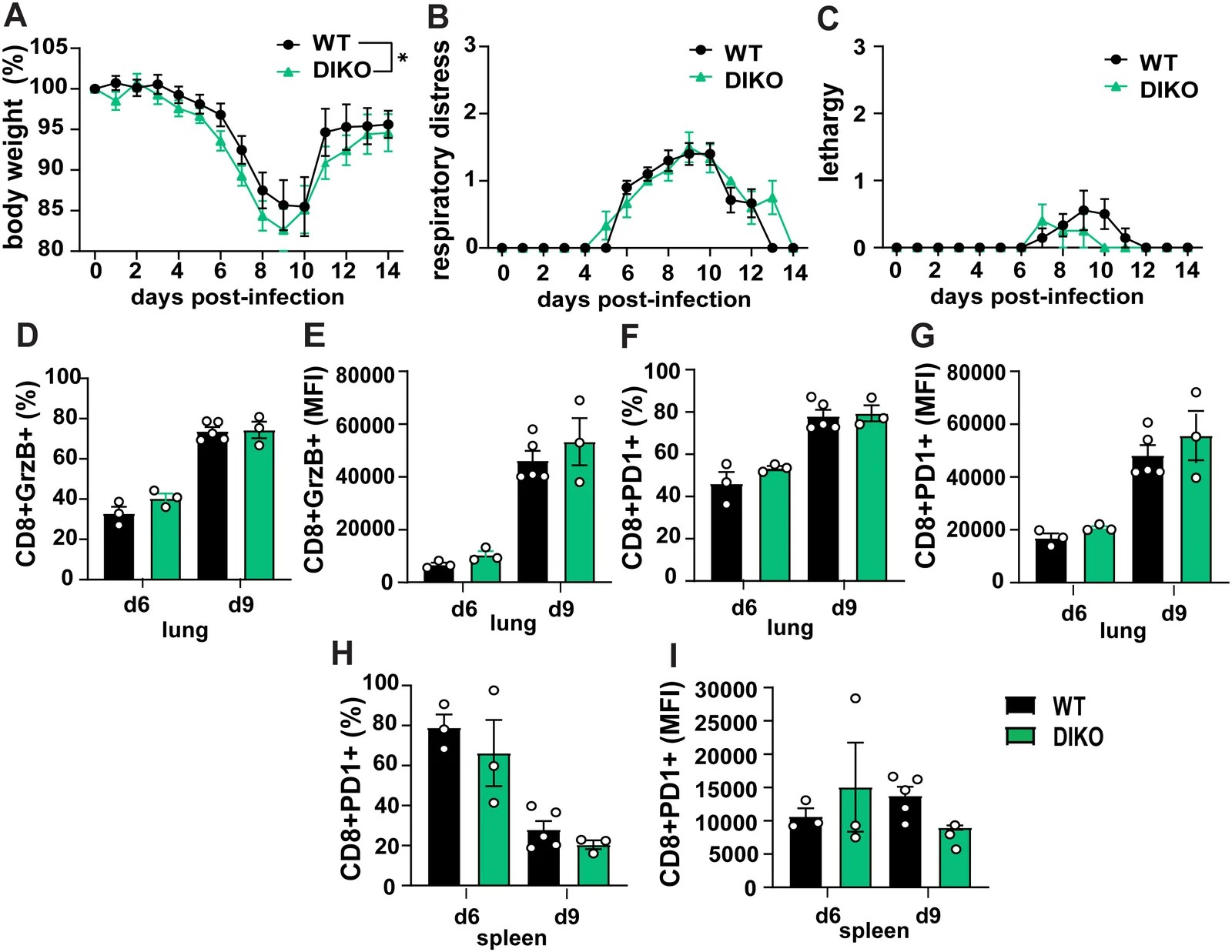
Renal Cyp27b1 is dispensable for host defense against influenza A virus infection. **(A)** Bodyweight loss, **(B)** respiratory distress and **(C)** lethargy scores following influenza A virus infection of DIKO (n-6) and WT littermates (n=10). **(D)** Frequency of CD8+/GrzB+, **(E)** GrzB MFI, and **(F)** frequency of CD8+/PD-1+ and **(G)** PD-1 MFI on CD8+ T cells in the lung. **(H)** Frequency of CD8+/PD-1+, and **(I)** PD-1 MFI on CD8+ T cells in the spleen. n=3–4 mice/group/timepoint and 1 experiment. Each symbol represents an individual mouse per timepoint. Values are the mean ± SEM. Significance was evaluated by two-way ANOVA with Tukey correction, *p < 0.05.

### 3.5 T cell expression of CYP27B1 and VDR are essential for optimal host response to influenza A virus

Cyp27b1 or Vdr were knocked out in T cells that express LCK and then infected with influenza A virus. Confirming the T cell specific KO of Cyp27b1, purified splenic T cells from WT mice expressed significantly more *Cyp27b1* mRNA than the T-Cyp27b1 KO T cells (**Supplementary Figure 5A**). Influenza A virus infection of T-Cyp27b1 KO mice and their WT littermates or T-Vdr KO mice and their WT littermates did not result in lethality. T-Cyp27b1 KO mice and T-Vdr KO mice lost significantly more bodyweight and suffered significantly more respiratory distress and lethargy than their respective WT littermates (**Figures 5A–F**). There were more CD8+ T cells at d6 post-infection and more CD4+ T cells at d9 post-infection in the lungs of T-Cyp27b1 KO mice than WT mice (**Figure 6A**; **Supplementary Figure 5B**). CD8+ T cells from T-Cyp27b1 KO mice had decreased GrzB expression compared to WT mice at d6 and d9 post-infection (**Figures 6B, C**). It has been previously shown that GrzB expression was reduced in CD8+ T cells from VDR KO mice compared to WT mice (26). CD8+ T cells in the lung of T- Cyp27b1 KO mice had lower PD-1 expression at d9 post-infection than CD8+ T cells from the WT lung (**Figures 6D, E**). Similarly, CD4+/PD-1+ expression was decreased in the lung of T- Cyp27b1 KO mice at d6 and d9 post-infection compared to WT mice (**Supplementary Figures 5C, D**). In the spleen PD-1 expression on CD8+ and CD4+ T cells from WT mice was significantly higher at d6 post-infection than on T cells from T-Cyp27b1 KO mice (**Figures 6F, G**; **Supplementary Figures 5E, F**). T cell intrinsic expression of CYP27B1 and the VDR are both required to control the severity of influenza A virus clinical disease in mice. Together, the data demonstrates a need for CD8+ T cells to produce CYP27B1 to support optimal GrzB+. The absence of either CYP27B1 or VDR expression in T cells results in significantly more severe influenza A virus disease as compared to WT littermates.

**Figure 5:**
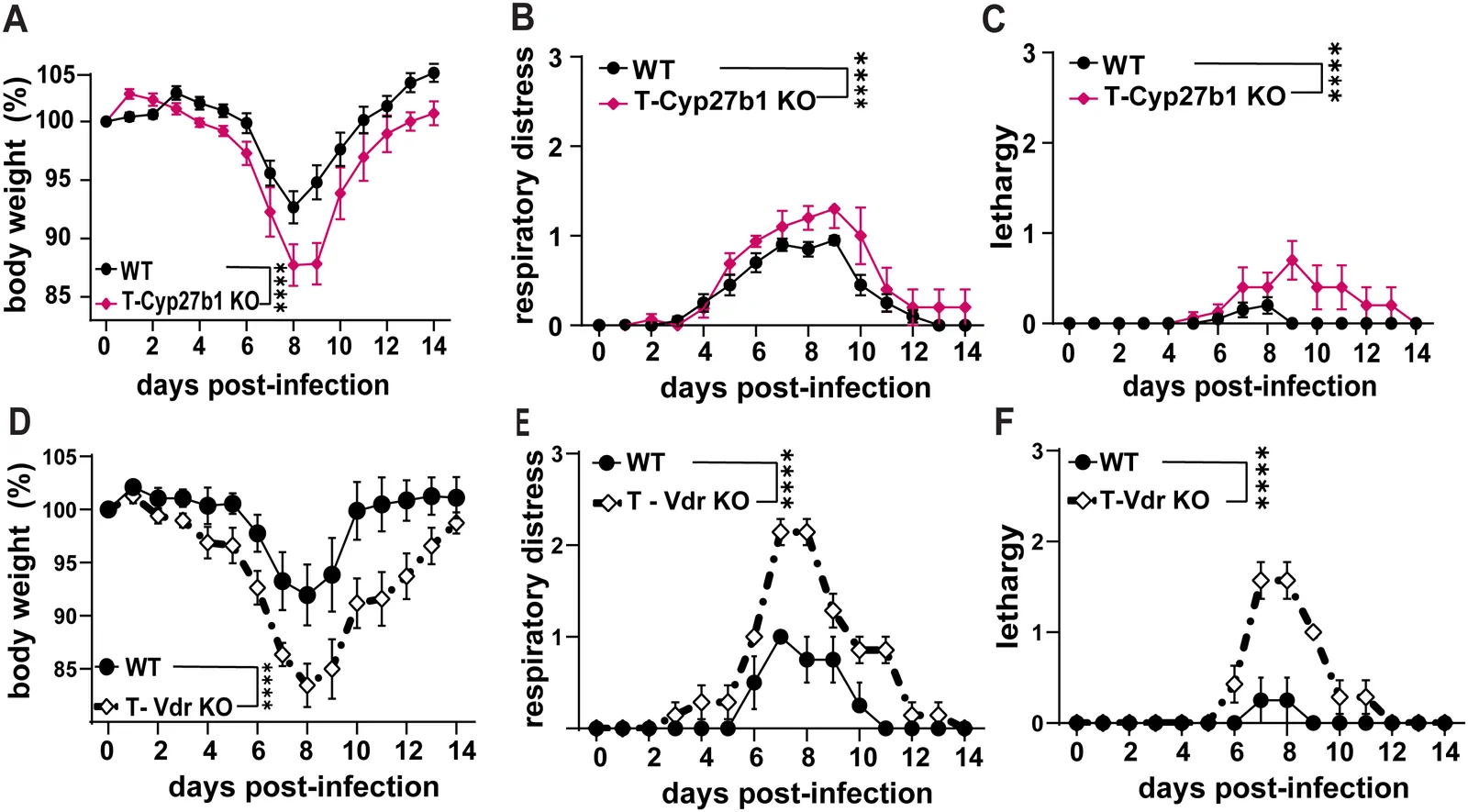
T cell Cyp27b1 and Vdr are required for an effective CD8+ T cell response. **(A, D)** Bodyweight loss, **(B, E)** respiratory distress, and **(C, F)** lethargy following influenza A virus infection of WT (=20), T-Cyp27b1 KO (n=16) and WT (n=4), T-Vdr KO (n=7) mice. WT and T-Cyp27b1 KO values are from 3 independent experiments and WT and T-Vdr KO values are from 1 experiment. Values are the mean ± SEM. Significance was analyzed by two-way ANOVA with Tukey’s multiple comparisons test and were adjusted using the two-stage step-up Benjamini, Krieger, and Yekutieli FDR for multiple comparisons, ****p < 0.0001.

**Figure 6:**
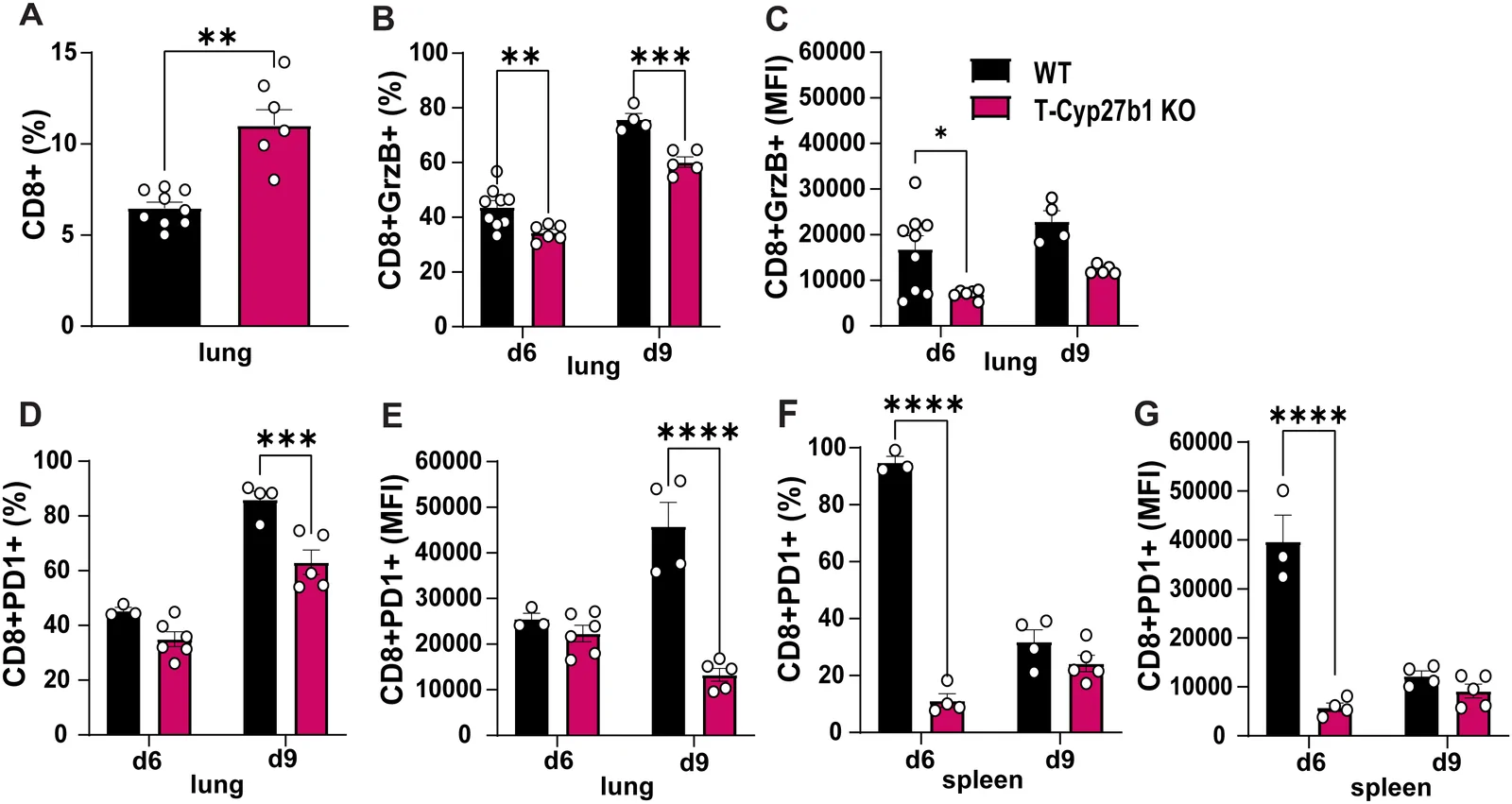
T cell Cyp27b1 production is required for an effective CD8+ T cell response. **(A)** Frequency of CD8+ T cells in the lung of WT (n=9) and T- Cyp27b1 KO (n=6) at d6 post-infection. **(B, C)** Frequency of CD8+/GrzB+ T cells and GrzB MFI in CD8+ T cells in the lung at d6 and d9 post-infection in WT (n=3-9) and T- Cyp27b1 KO mice (n=5-6). **(D, E)** Frequency of CD8+/PD-1+ T cells and PD-1 MFI on CD8+ T cells in the lung. **(F, G)** Frequency of CD8+/PD-1+ T cells and PD-1 MFI on CD8+ T cells in the spleen at d6 and d9 of WT (n=3-4) and T- Cyp27b1 KO (n=4-5) mice. Each symbol represents an individual mouse per timepoint. Values are the mean ± SEM and are based on 3 independent experiments. Significance was evaluated by two-way ANOVA with Tukey’s multiple comparisons test and were adjusted using the two-stage step-up Benjamini, Krieger, and Yekutieli FDR for multiple comparisons, *p < 0.05, **p < 0.01, ***p < 0.001, ****p < 0.0001.

## 4 Discussion

Identifying the source of the 1,25(OH)_2_D that regulates the vitamin D response of immune cells is a critical unanswered question in vitamin D immunobiology. The ability of a mouse to produce any 1,25(OH)_2_D is critical for resistance to influenza, as the whole body Cyp27b1 KO mice developed lethal severe respiratory illness following infection. DIKO mice developed respiratory distress and lethargy that was not significantly different from WT mice; suggesting that endocrine (kidney-derived) 1,25(OH)_2_D is not essential for host immunity to influenza. Multiple extra-renal cell types have been shown to be potential sources of 1,25(OH)_2_D, including lung epithelial cells, macrophage, and T cells (36). The data here provide the first example of an *in vivo* role for T cell production of CYP27B1 in regulating the local immune response to an influenza virus. T-Cyp27b1 KO mice showed significantly more severe influenza symptoms than WT mice. T cells only express CYP27B1 and produce 1,25(OH)_2_D upon activation (25, 36). We did not measure the amount of 1,25(OH)_2_D produced by primary T cells from the lung of our mice, which is a limitation. However, the data support a model where T cells that produce 1,25(OH)_2_D via CYP27B1 are important for effective host immunity to an H1N1 strain of influenza A virus.

Cytotoxic CD8+ T cells are critical to the host immune response against influenza A virus. The finding that there are fewer CD8+ Grzb expressing T cells and less Grzb+ expressed per CD8+ T cell in the whole body Cyp27b1 KO and T-Cyp27b1 KO mice as compared to WT littermates, suggests that the diminished GrzB+ CD8+ T cell response is associated with the increased severity of influenza in the Cyp27b1 and T-Cyp27b1 KO mice to influenza infection. Whole body Cyp27b1 KO mice have altered mineral homeostasis and bone effects not present in the T-Cyp27b1 KO mice. The data that show that deficiency in the T cell enzyme that produces 1,25(OH)_2_D is connected to robust GrzB expansion is novel and suggests a role for autocrine production of 1,25(OH)_2_D that supports GrzB production in CD8+ T cells. VDR KO mice also have been shown to have CD8+ T cells with lower GrzB expression following viral infection with lymphocytic choriomeningitis virus (26). In a tumor model, vitamin D supplementation to already vitamin D sufficient mice resulted in increased frequencies of CD8+/GrzB+ and CD8+/PD-1+ T cells compared to the vitamin D sufficient control mice (37). A positive correlation between circulating 25(OH)D levels and influenza A virus specific GrzB expression in humans that received an influenza A virus vaccine has also been shown (38). Vitamin D response elements (VDRE) in the GrzB gene in mice or humans have not been identified. It may be that production of CYP27B1 by T cells regulates GrzB indirectly. The inability of *in vivo* 1,25(OH)_2_D treatments to induce GrzB+ expression in CD8+ T cells from Cyp27b1 KO mice, suggests that the effect of Cyp27b1 KO on GrzB+ expression may be through epigenetic effects and irreversible. Vitamin D has been implicated as an epigenetic regulator of several immune cells, including invariant NK cells, regulatory T cells, and hematopoietic stem cells (HSC) (39–41). Alternatively, Cyp27b1 KO mice have been shown to have fewer HSC than WT mice early during embryonic development (19). Therefore, a developmental effect of Cyp27b1 KO on CD8+ T cells cannot be ruled out by the current study. In addition, the LCK promoter used to delete CYP27B1 in T cells is expressed early in thymic development. Future experiments with an inducible deletion of CYP27B1 would be needed to determine why the 1,25(OH)_2_D treatments of the Cyp27b1 KO mice did not restore CD8+/GrzB+ T cells and whether the developmental effects of 1,25(OH)_2_D deficiency are reversible with prolonged supplementation. None-the-less the data presented here suggest an important role for the CYP27B1 enzyme as an autocrine source of 1,25(OH)_2_D that supports CD8+/GrzB+ T cells.

PD-1 is an activation marker on T cells, which is induced following infection and then decreases with virus clearance (42). Following influenza A virus infection, PD-1 expression is increased on lung and splenic T cells to regulate the host immune response and to prevent immunopathology. PD-1 expression was significantly lower on CD4+ and CD8+ T cells in the spleen of Cyp27b1 KO mice at d6 post-influenza A virus infection. Vitamin D is an inducer of the PD-1/PD-L1 pathway (43). Following influenza A virus infection, effector CD8+ T cells increase expression of PD-1 that is critical for memory T cell development following viral infection (42, 44). During acute influenza A virus infection, T cell activation drives the rapid upregulation of PD-1. The induction of PD-1 acts as an inhibitory checkpoint to prevent excessive tissue damage and mediate homeostasis. Cyp27b1 KO T cells proliferated more quickly and produced more IFN-γ than WT T cells. The data is consistent with a link between CYP27B1 and the autocrine production of 1,25(OH)_2_D that coincides with the correlative changes in PD-1 and more inflammation following influenza infection. More research is needed into blocking the PD-1/PD-1L pathway to determine the mechanism of how local production of 1,25(OH)_2_D by T cells shapes PD-1 expression during influenza infection.

There are several limitations to the current study. First, the experiments were conducted using only a single influenza A virus strain. Because influenza viruses are highly diverse, constantly mutating, and capable of interacting with the host immune system in strain-specific ways, relying on a single strain may limit the broad generalizability of our findings. Conversely, utilizing a single, predictable influenza strain was essential for minimizing confounding viral variables; this approach provided a standardized baseline that allowed us to directly compare host-pathogen dynamics across multiple distinct genetic models. Second, because vitamin D impacts the development of the primary lymphoid organs (thymus and bone marrow) and the structural integrity of the lung epithelium, it is incredibly difficult to separate a baseline immune defect from the systemic, metabolic sickness of the full Cyp27b1 KO mouse. DIKO mice still possess the extra-renal capacity to express CYP27B1 which preserves baseline calcium homeostasis and prevents bone deformities, with an uncompromised baseline immune system. The data show normal numbers of CD8+ GrzB+ T cells in the DIKO lung and no significant difference in the symptoms of infection compared to WT mice demonstrating the importance of extra-renal production of 1,25(OH)_2_D on CD8+ T cells and the symptoms following influenza A infection. Third, local 1,25(OH)_2_D production was not directly quantified inside the CD8+ T cells or within their immediate cell culture supernatants. While CYP27B1 expression and downstream VDR activation serve as reliable proxies for vitamin D metabolism, direct confirmation of the enzymatic end-product was omitted. The active hormone 1,25(OH)_2_D is a highly potent molecule that functions at low concentrations and with a short half. The data that show alterations to T cell function in the absence of CYP27B1 in T cells, both *in vivo* and *in vitro*, suggest that the WT T cells that express *Cyp27b1* mRNA following activation are able to produce local 1,25(OH)_2_D that is responsible for the differences between T-Cyp27b1 KO and WT responses to influenza infection.

The ability of T cells and other extra-renal immune and non-immune cells to produce 1,25(OH)_2_D was associated with alterations to the CD8+ T cell response that is correlated with increased severity of influenza A virus disease *in vivo*. Expression of CYP27B1 by CD8+ T cells is important for GrzB expression and effective cytotoxic T cell numbers could not be recovered by systemic supplementation of Cyp27b1 KO mice with 1,25(OH)_2_D at the time of infection. In addition, Cyp27b1 expression in T cells is important for the support of GrzB+ expressing CD8+ T cells. The work expands our understanding of the mechanisms by which vitamin D regulates the host immune response to influenza A virus infection. The data demonstrates that extra-renal CYP27B1 and especially T cell intrinsic production of CYP27B1 is an important determinant of the vitamin D mediated anti-viral immune response. Future research may examine strategies to enhance CYP27B1 activity in immune cells as a target for reducing severity of influenza virus disease. These findings reframe our understanding of how vitamin D exerts its protective effects following respiratory infection, shifting the focus from systemic circulating levels to local, cell-intrinsic vitamin D signaling within the immune microenvironment.

## Data Availability

The raw data supporting the conclusions of this article will be made available by the authors, without undue reservation.
