# Supplementary material for: T cell Cyp27b1 promotes granzyme B expression and antiviral host defense in a mouse model of H1N1 influenza A virus infection
Source: Front Immunol. 2026 Oct 14;17:1944713. doi: 10.3389/fimmu.2026.1944713 (PMC13713429; doi:10.3389/fimmu.2026.1944713)
Supplement: Supplementary file 1 [file DataSheet1.pdf]

Supplementary Tables

STable 1. Genotyping Primers

	Forward Primer (5'-3')	Reverse Primer (5'-3')	Band Size
Cyp27b1 WT	GTTCCACTGCCCCGG GTCAA	CAGCGCCATGCACCT AGAGA	WT: 451 bp
Cyp27b1 KO	GCGTAATAGCGAAGA GGCCCGC	GTGAGCGAGTAACAA CCCGTCGG	KO: 270 bp
Cyp27b1 Flox	GGTGAACAGCGAGT GTAGGAGCTAAGTTG	GAAGGAGGGCAGATT AGATATTCTAGGATGC	WT: 352 bp<br>Flox: 465 bp
LCK WT	CTAGGCCACAGAATT GAAAGATCT	GTAGGTGGAAATTCTA GCATCATCG	WT: 465 bp
LCK Cre	CCTCCTGTGAACTTG GTGCTTGAG	TGCATCGACCGGTAAT GCAG	Cre: 200 bp
VDR Flox	ATG GAC AGG AAC ACA CAG CAT CA	TCT GAC TCC CAC AAG TGT ACC ACG G	WT: 254 BP<br>Flox: 337 bp

STable 2. Flow Cytometry

Target- Fluorophore	Clone	Manufacturer, Catalog #
CD3- AF647	17A2	BIOLEGEND, 100209
CD4- BV421	GK1.5	BIOLEGEND, 100437
CD8α – PECY7	53-6.7	BIOLEGEND, 100722
GRANZYME B - FITC	GB11	BIOLEGEND, 515403
CD279 PD-1 – PE	29F.1A12	BIOLEGEND, 135205

Cell Type	Markers
CD4+	CD3+CD8-CD4+
CD8+	CD3+CD4-CD8+
CD4+GrzB+	CD3+CD8-CD4+GrzB+
CD8+GrzB+	CD3+CD4-CD8+Grzb+
CD4+PD1+	CD3+CD8-CD4+PD1+
CD8+PD1+	CD3+CD4-CD8+PD1+

STable. 3 qPCR

Target Gene	Forward Primer (5'-3')	Reverse Primer (5'-3')
Cyp24a1	5'-ACC CCC AAG GTC CGT GAC ATC-3	5'-CCA GTT GGG TCC AGG TAA GG-3'
H1N1 *M GENE*	5'-AGA TGA GTC TTC TAA CCG AGG TCG-3'	5'-TCG AGA TCG GTG TTC TTT CC-3'
Cyp27b1	5'-TCT ATG AGC TTT CCC GGC ACC CC-3′	5'-GTC ATG GGC TTG ATA GGA AGA G-3′

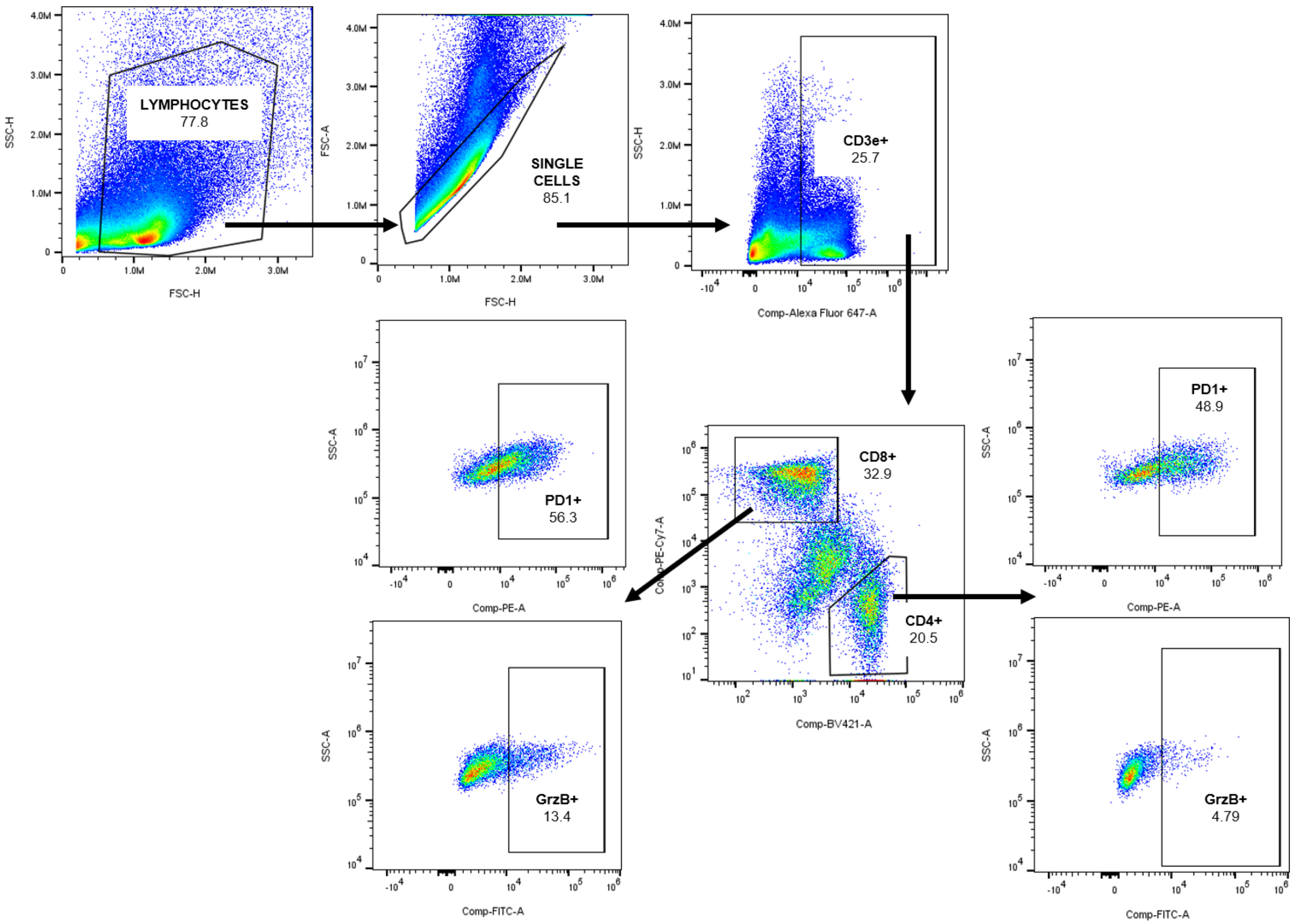

Supplementary Figure 1. Gating strategy for granzyme B and PD1+ on CD4+ and CD8+ T cells. Gating on lymphocytes, then singlets, and then CD3e+ for T cells. T cells are then differentiated between CD4+ or CD8+ populations, and within these populations PD1+ and GrzB+ positive cells are identified.

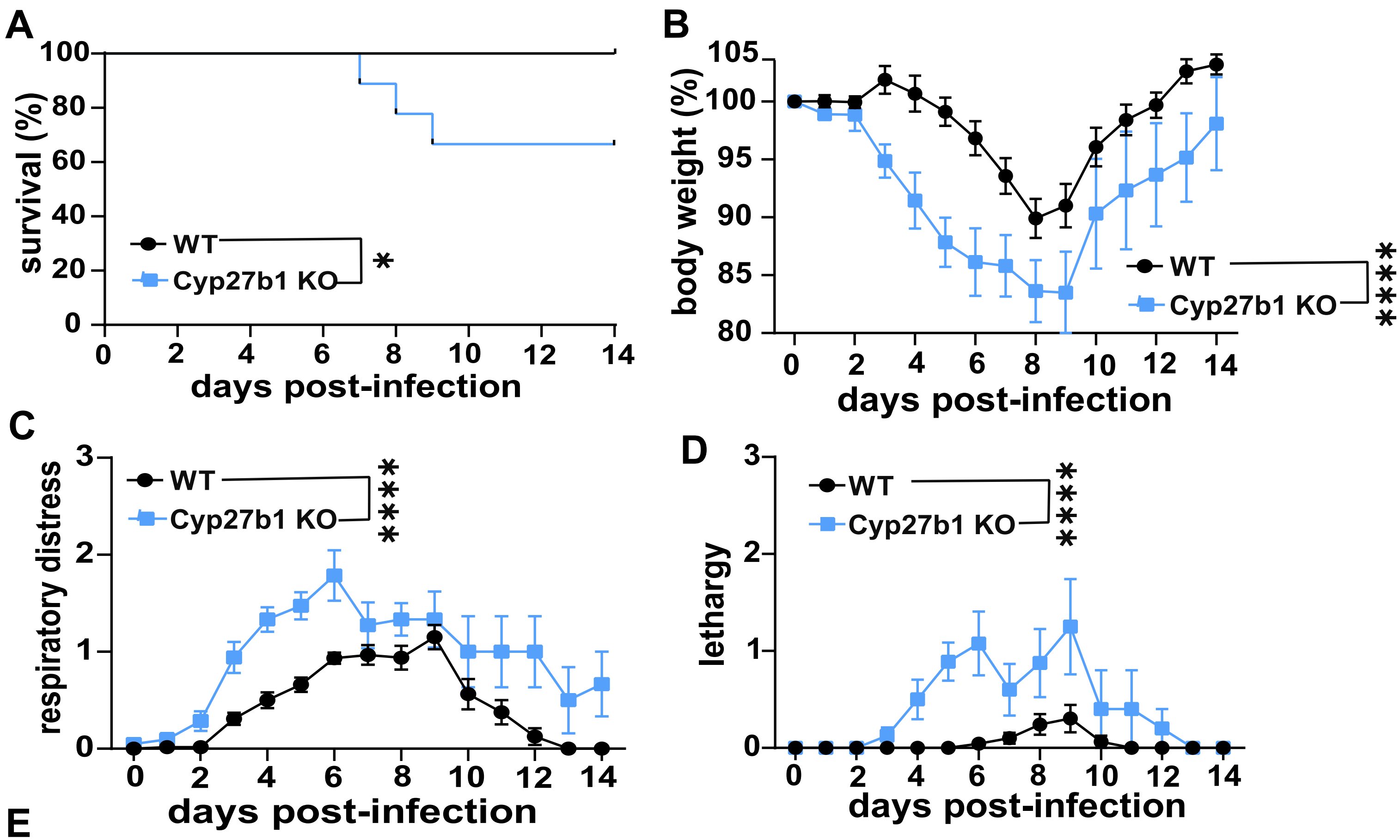

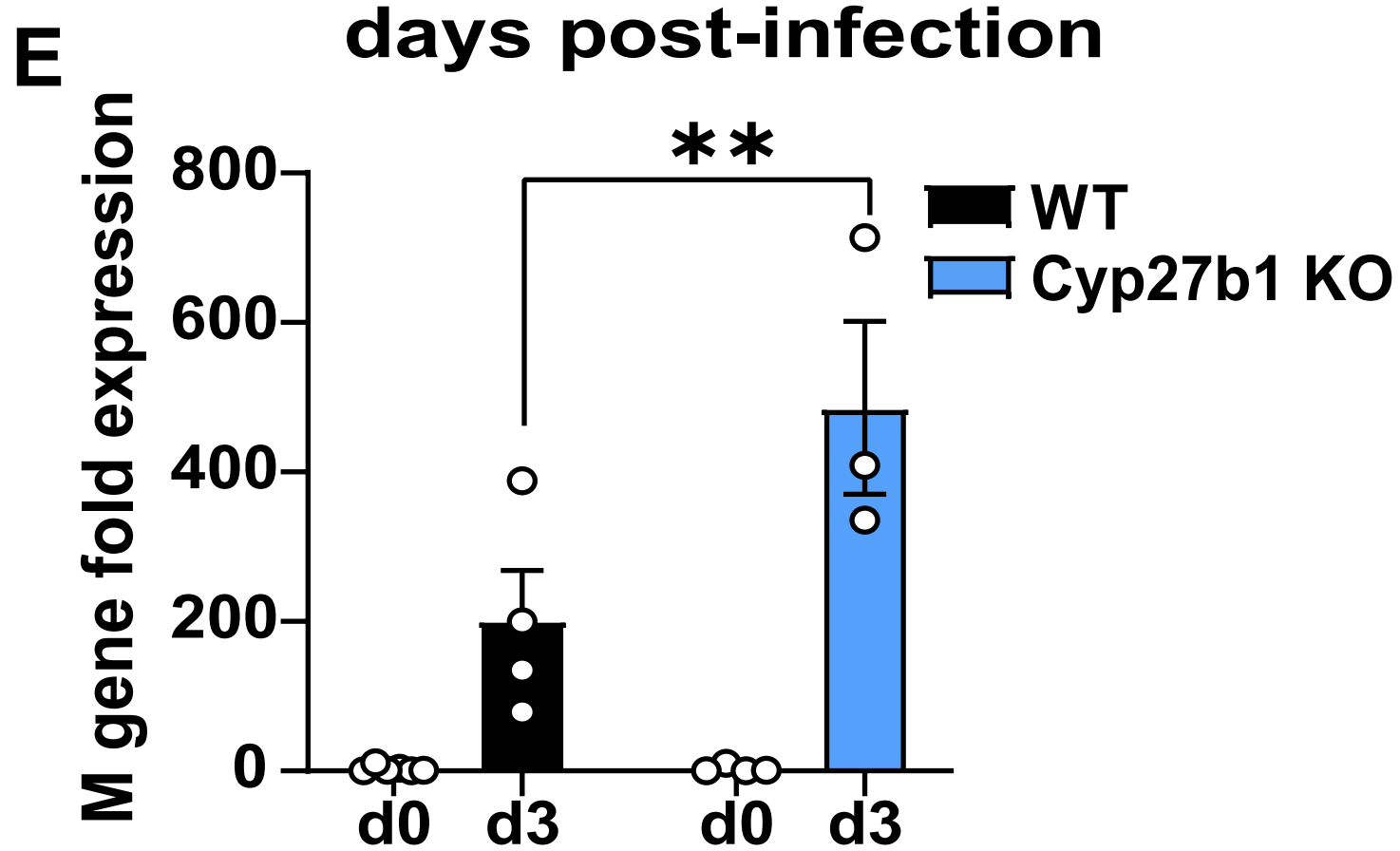

SFig 2. Cyp27b1 KO results in increased lethality, influenza-induced distress, and higher viral M gene expression.
WT and Cyp27b1 KO mice were monitored for (A) survival, (B) bodyweight, (C) respiratory distress and (D) lethargy scores. (E) Viral M gene in lung tissue. n=3-5 mice per time point per group. Values are the mean ± SEM. Signifcance was evaluated by (A) Mantel-Cox log-rank test and (B-E) two-way ANOVA with Tukey's multiple comparisons test and were adjusted using the two-stage step-up Benjamini, Krieger, and Yekutieli FDR for multiple comparisons, *$p < 0.05$, **$p < 0.01$, ****$p < 0.0001$.

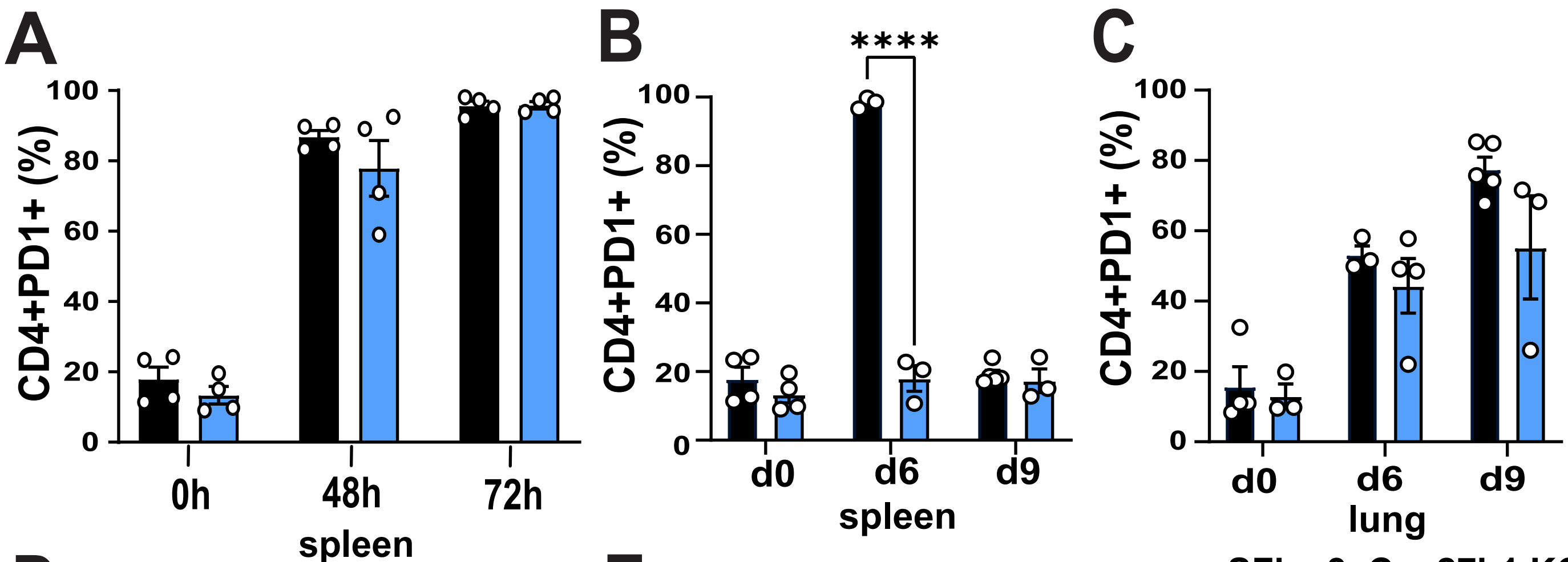

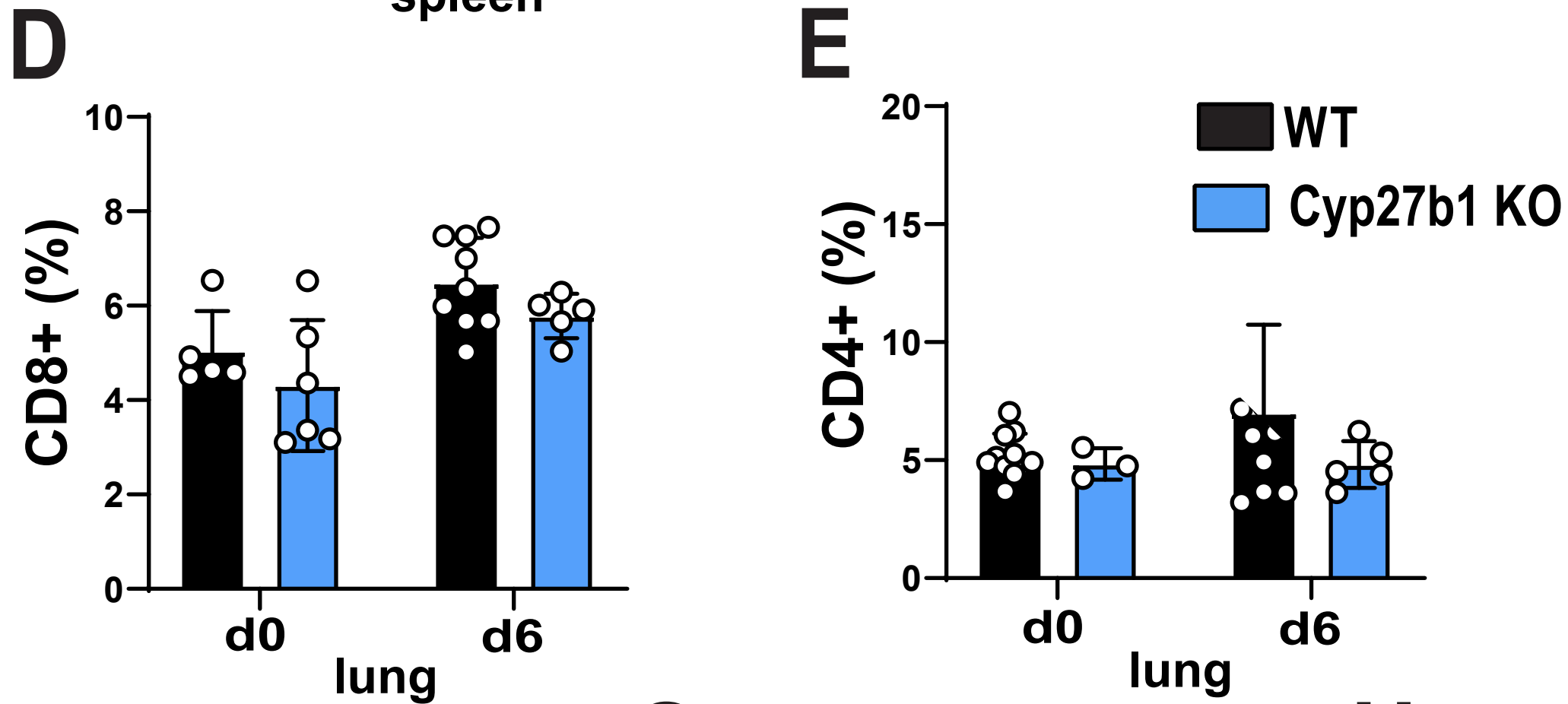

SFig. 3 Cyp27b1 KO CD4+ T cells fail to upregulate PD-1 in the spleen. Frequency of CD4+PD1 in (A) splenocytes following activation in vitro (n=4 per group), or (B) in the spleen and (C) lung following influeza infection. Frequency of (D) CD8+ and (E) CD4+ T cell frequency. MFI of PD-1+ in the spleen (F) and lung (G). (H,I) (H) Frequency of GrzB+ and (I) GrzB MFI. B-I n=3-8 mice per group per timepoint. Values are the mean ± SEM. Statistical significance was determined by (A) unpaired Student's t-test or (B-I) two-way ANOVA with Tukey correction (multi-group/timepoint comparisons), ****p < 0.0001.

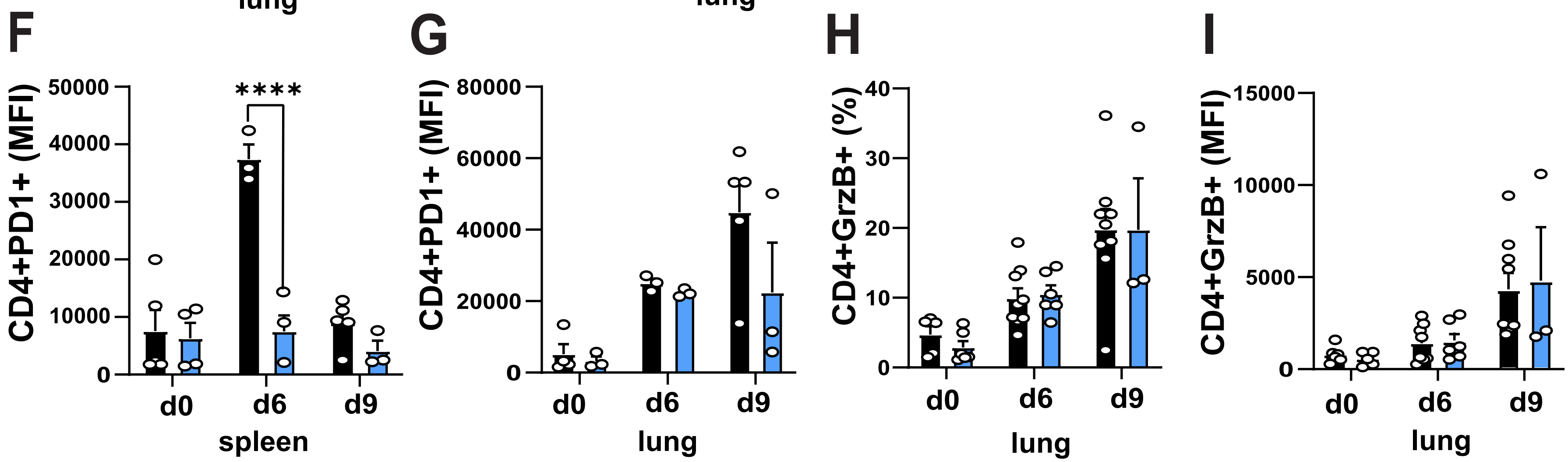

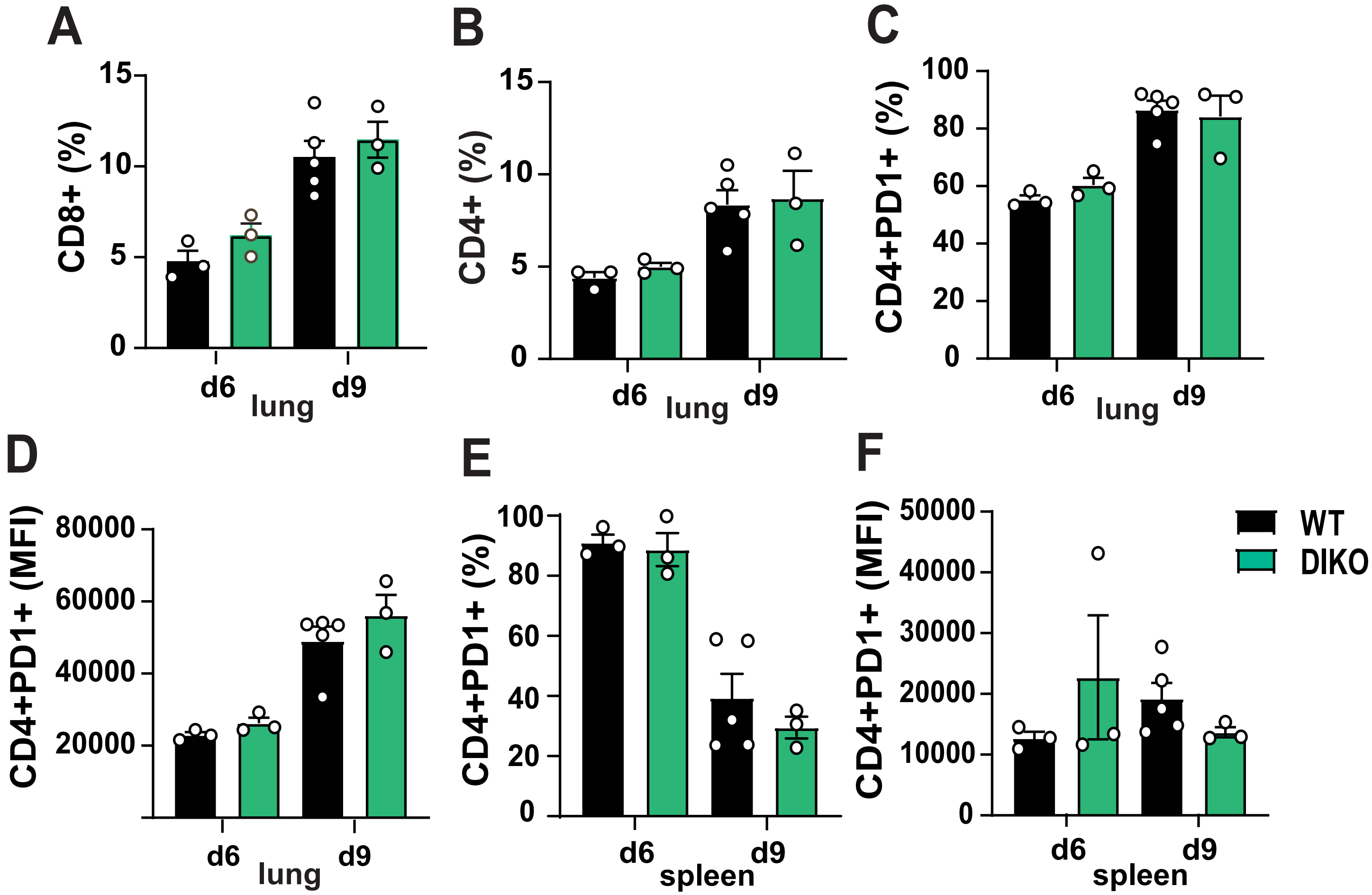

SFig. 4 PD1 expression on CD4+ T cells following influenza infection. Frequency of (A) CD8+ T cells and (B) CD4+ T cells in the lung following influenza infection. Frequency of (C,E) CD4+PD-1+ T cells and (D,F) PD-1 MFI on CD4+ T cells in the (A-D) lung and spleen (E,F), n=3-5 mice per group and per timepoint. Values are the mean ± SEM. Statistical signficiance A-F) used two-way ANOVA with Tukey correction.

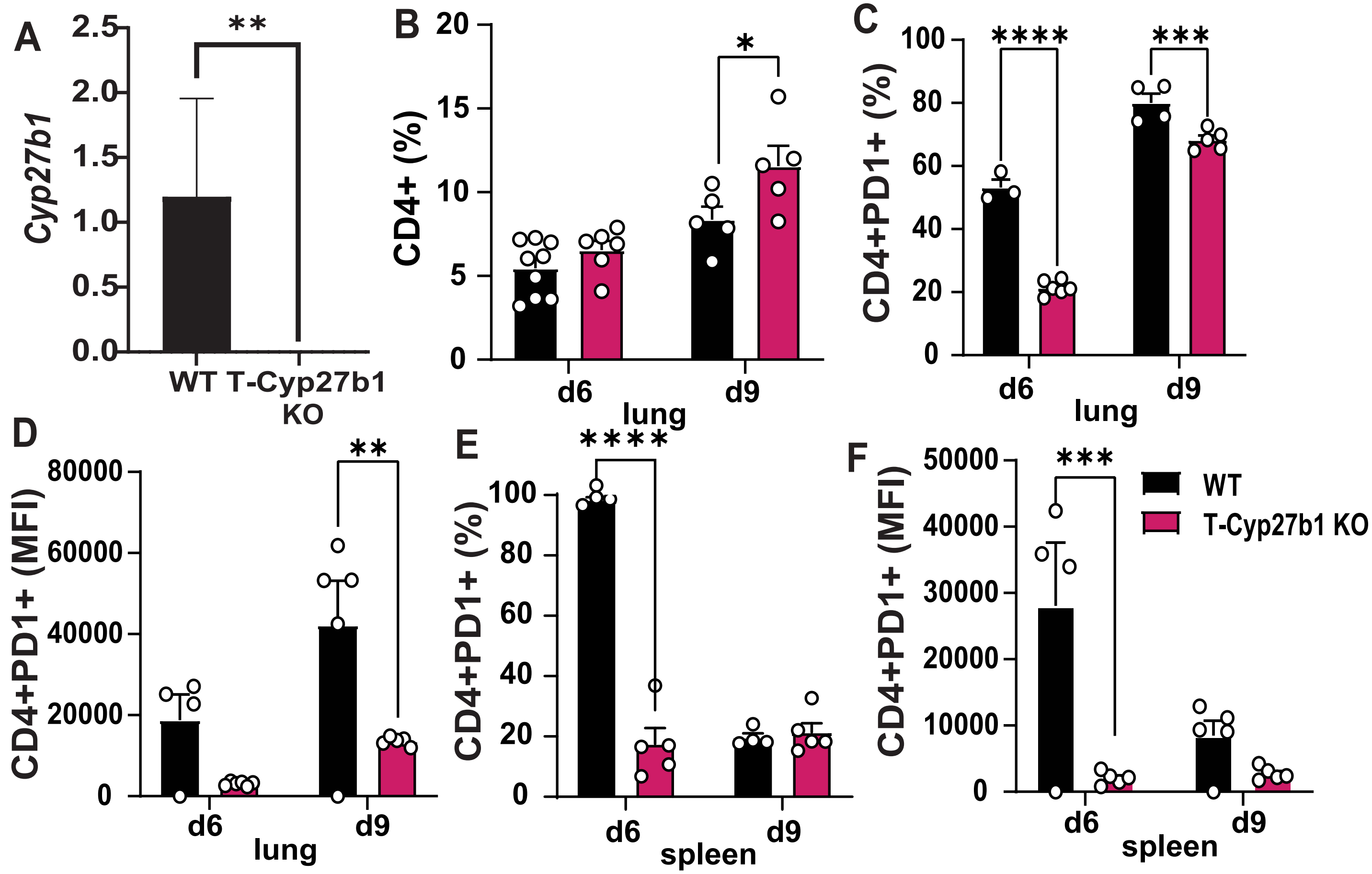

SFig. 5 T cell 1,25(OH)$_2$D affects PD1 expression on CD4+ T cells. (A) *Cyp27b1* mRNA expression from purified splenic T cells. Frequency of (B) CD4+ T cells (C) CD4+PD1+, and (D) PD1+ MFI on T cells from the lung. Frequency of (E) CD4+PD1+ T cells and (F) PD1 MFI on CD4+ T cells in the spleen. Values are the mean ± SEM. Statstical significance was by two-way ANOVA with Tukey correction, n=4-5 mice per group per timepoint, *p < 0.05, **p < 0.01,***p < 0.001, ****p < 0.0001.
